# Crystal structure of 2,7-dieth­oxy-1,8-bis­(4-nitro­benzo­yl)naphthalene

**DOI:** 10.1107/S1600536814018674

**Published:** 2014-08-23

**Authors:** Saki Mohri, Shinji Ohisa, Keiichi Noguchi, Noriyuki Yonezawa, Akiko Okamoto

**Affiliations:** a Department of Organic and Polymer Materials Chemistry, Tokyo University of Agriculture & Technology (TUAT), Koganei, Tokyo 184-8588, Japan; b Instrumentation Analysis Center, Tokyo University of Agriculture & Technology (TUAT), Koganei, Tokyo 184-8588, Japan

**Keywords:** crystal structure, *peri*-aroyl­naphthalene, naphthalene diketone, non-coplanarly accumulated aromatic-rings structure, spatial organization

## Abstract

The title compound, C_28_H_22_N_2_O_8_, possesses crystallographically imposed twofold symmetry, with the two central carbon atoms of the naphthalene unit lying on the rotation axis. The two benzoyl groups in the mol­ecule are twisted away from the attached naphthalene unit with a C—C—C=O torsion angle of 49.05 (15)° between the naphthalene unit and the carbonyl group. The dihedral angle between the naphthalene ring system and the benzene ring is 77.17 (5)°. In the crystal, pairs of C—H⋯O=C hydrogen bonds link the mol­ecules into a double-column structure along the *c* axis. The mol­ecules are further linked by C—H⋯O=N hydrogen bonds, forming a three-dimensional network. C—H⋯π inter­actions between the methyl­ene group and the naphthalene unit and π–π inter­actions between the naphthalene ring systems [centroid–centroid distances of 3.7858 (7)–3.7860 (7) Å] are also observed.

## Abstract 2

The title compound possesses crystallographically imposed twofold symmetry with two C atoms lying on the rotation axis and has a non-coplanarly accumulated aromatic-rings structure. In the crystal, C—H⋯O=C hydrogen bonds between the benzene rings and the carbonyl groups and C—H⋯O=N hydrogen bonds between the benzene rings and the nitro groups are observed; these C—H⋯O hydrogen bonds link the mol­ecules, forming a three-dimensional structure.

## Chemical context   

Mol­ecules with non-coplanarly accumulated aromatic rings, such as biphenyl and binaphthyl derivatives, have been in the limelight as unique building blocks affording characteristic optical and electronic properties (Hatano *et al.*, 2013 ▶; Park *et al.*, 2010 ▶; Vaghi *et al.*, 2013 ▶) and asymmetric mol­ecular environments (Bulman Page *et al.*, 2012 ▶; Jayalakshmi *et al.*, 2012 ▶; Kano *et al.*, 2006 ▶; Wang *et al.*, 2014 ▶). *peri*-Substituted naphthalenes have also much attention as characteristic aromatic ring core compounds and the structural analyses have been actively performed (Cohen *et al.*, 2004 ▶; Jing *et al.*, 2005 ▶).
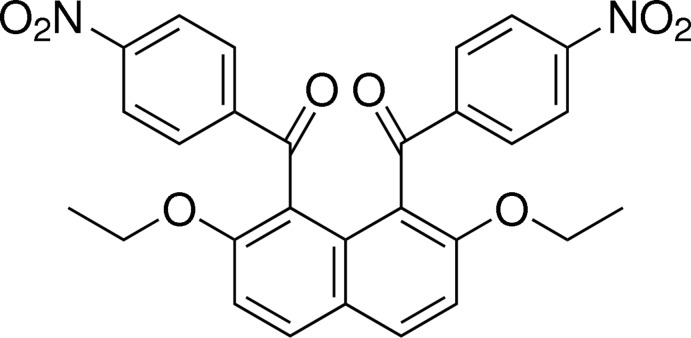


In the course of our study on selective electrophilic aromatic aroylation of 2,7-di­meth­oxy­naphthalene, *peri*-aroyl­naphthalene compounds have proved to be formed regio­selectively with the aid of a suitable acidic mediator (Okamoto & Yonezawa, 2009 ▶; Okamoto *et al.*, 2011 ▶). According to X-ray crystal analysis, the resulting 1,8-diaroyl­naphthalene and 1-monoaroyl­naphthalene compounds have non-coplanarly accumulated aromatic-ring structures in their crystals. The aroyl groups at the 1,8-positions (or 1-position) of the naphthalene ring system in the mol­ecules are situated in a perpendicular fashion to the naphthalene ring system, as shown in 1,8-dibenzoyl-2,7-di­meth­oxy­naphthalene (Nakaema *et al.*, 2008 ▶), 1,8-dibenzoyl-2,7-di­eth­oxy­naphthalene (Isogai *et al.*, 2013 ▶) and 1-(4-nitro­benzo­yl)-2,7-di­meth­oxy­naphthalene (Watanabe *et al.*, 2010 ▶). As a part of our continuous study on the mol­ecular structures of this kind of mol­ecules, the X-ray crystal structure of the title compound is reported here.

## Structural commentary   

**Figure 1:**
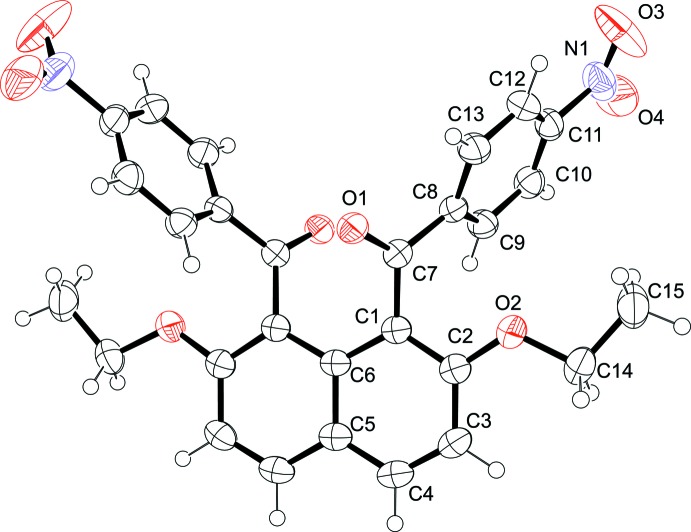
The mol­ecular structure of the title compound with the atom numbering. The displacement ellipsoids are drawn at the 50% probability level.

The title mol­ecule lies on a crystallographic twofold axis running through atoms C5 and C6 of the naphthalene unit so that the asymmetric unit consists of one half-mol­ecule (Fig. 1 ▶). In the mol­ecule, two aroyl groups are non-coplanarly attached to the naphthalene ring system. The torsion angles of the benzene ring and the naphthalene ring system with the ketonic carbonyl moiety (O1—C7—C8—C13 and C6—C1—C7—O1) are 26.83 (17) and 49.05 (15)°, respectively. The dihedral angle between the benzene ring and the naphthalene ring system is 77.17 (5)°. The benzene ring and the nitro group are approximately coplanar with a dihedral angle of 5.0 (2)°.

## Supra­molecular features   

**Figure 2:**
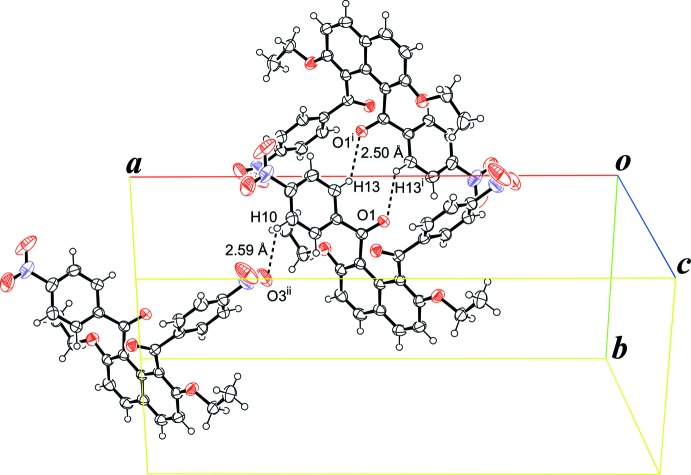
A crystal packing view of the title compound, showing the inter­molecular C—H⋯O=C and C—H⋯O=N inter­actions. [Symmetry codes: (i) 1 − *x*, −*y*, −*z*; (ii) 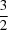 − *x*, 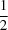 + *y*, 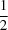 − *z*.]

**Figure 3:**
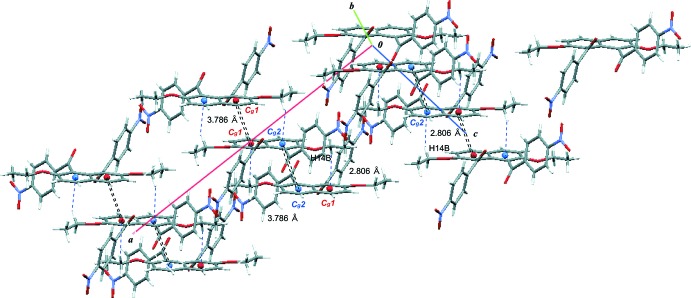
A crystal packing view of the title compound, showing the inter­molecular C—H⋯π inter­actions (dashed lines) and π–π inter­actions (double dashed lines).

In the crystal, the mol­ecular packing of the title compound is mainly stabilized by a pair of C—H⋯O=C hydrogen bonds between the benzene ring and the ketonic carbonyl group (C13—H13⋯O1^i^; details and symmetry code in Table 1 ▶), which make an 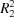
(10) ring motif (Fig. 2 ▶). The mol­ecules are stacked through these inter­actions in a double-column along the *c* axis. The mol­ecules are also linked by C—H⋯O=N inter­actions between the benzene ring and the nitro group (C10—H10⋯O3^ii^; Table 1 ▶), forming a three-dimensional network and thus resulting in the inter­penetration of the naphthalene unit into the adjacent double-column. π–π inter­actions between the inter­penetrating naphthalene ring systems are observed; the inter­planar distance is 3.5326 (4) Å and the centroid–centroid distances are 3.7860 (7), 3.7859 (7) and 3.7858 (7) Å, respectively, for *Cg*1⋯*Cg*1^vi^, *Cg*1⋯*Cg*2^vii^ and *Cg*2⋯*Cg*2^viii^, where *Cg*1 and *Cg*2 are the centroids of the six-membered C1–C6 and C1^v^–C4^v^/C5/C6 rings, respectively [symmetry codes: (v) −*x* + 1, *y*, −*z* + 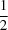; (vi) −*x* + 1, −*y* + 1, −*z*; (vii) *x*, −*y* + 1, *z* − 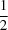; (viii) −*x* + 1, −*y* + 1, −*z* + 1]. C—H⋯π inter­actions between the methyl­ene group and the naphthalene ring system (C14—H14*B*⋯*Cg*1^iii^ and C14—H14*B*⋯*Cg*2^iv^; Table 1 ▶ and Fig. 3 ▶) are also observed.

## Database survey   

A search of the Cambridge Structural Database (Version 5.35, last update May 2014; Allen, 2002 ▶) showed 39 and 29 structures containing the 1,8-diaroyl­naphthalene (including 1,8-dialkanoyl­naphthalene) and 1,8-diaroyl-2,7-di­alk­oxy­naph­thal­ene units, respectively. The title compound has a non-coplanarly accumulated aromatic-rings structure, as found in the nitro group-free 1,8-di­benzoyl­naphthalene homologues and the nitro-group-bearing 1-benzoyl­naphthalene homologue, *viz.* 1,8-dibenzoyl-2,7-di­meth­oxy­naphthalene (Naka­ema *et al.*, 2008 ▶) and 1,8-dibenzoyl-2,7-di­eth­oxy­naphthalene (Isogai *et al.*, 2013 ▶), and 2,7-dimeth­oxy-1-(4-nitro­benzo­yl)naphthalene (Watanabe *et al.*, 2010 ▶). The dihedral angle between the benzene ring and the naphthalene ring system [77.17 (5)°] is close to those of the three homologues [68.42 (5) and 71.69 (5)° for 1,8-dibenzoyl-2,7-di­eth­oxy­naphthalene; 83.59 (5)° for 1,8-dibenzoyl-2,7-di­meth­oxy­naphthalene; 61.97 (5)° for 1-(4-nitro­benzo­yl)-2,7-di­meth­oxy­naphthalene]. On the other hand, the torsion angle between the carbonyl group and the benzene ring is different from the homologues, *i.e.*, the title compound [26.83 (17)°] > 1-(4-nitro­benzo­yl)-2,7-di­meth­oxy­naphthalene [−13.29 (17)°] >> 1,8-dibenzoyl-2,7-di­eth­oxy­naphthalene [1.58 (17)° and 1.44 (17)°] > 1,8-dibenzoyl-2,7-di­meth­oxy­naphthalene [0.4 (2)°]. Although the C—H⋯O=C inter­actions between the benzene ring and the ketonic carbonyl group are observed in all of four homologues, the C—H⋯O=N inter­action is observed only in the title compound.

## Synthesis and crystallization   

To a 10 ml flask, 4-nitro­benzoic acid (3.96 mmol, 735 mg), aluminium chloride (4.35 mmol, 580 mg) and methyl chloride (3.0 ml) were placed and stirred at 273 K. To reaction mixture thus obtained, 2,7-di­eth­oxy­naphthalene (0.6 mmol, 130 mg) was added. After the reaction mixture was stirred at 273 K for 48 h, it was poured into ice-cold water (10 ml). The aqueous layer was extracted with CHCl_3_ (10 ml×3). The combined extracts were washed with 2 *M* aqueous NaOH followed by washing with brine. The organic layers thus obtained were dried over anhydrous MgSO_4_. The solvent was removed under reduced pressure to give cake. The crude product was purified by reprecipitation (CHCl_3_/methanol) (isolated yield 27%). Finally, the isolated product was crystallized from CHCl_3_-hexane (*v*/*v* = 2:1) to give single crystals.

^1^H NMR (300 MHz, CDCl_3_): δ 0.91 (6H, *t*, *J* = 5.2 Hz), 3.98 (4H, *q*, *J* = 5.2 Hz), 7.18 (2H, *d*, *J* = 6.9 Hz), 7.91 (4H, *d*, *J* = 6.6 Hz), 8.00 (2H, d, *J* = 6.9 Hz), 8.26 (4H, *d*, *J* = 6.6 Hz); ^13^C NMR (75 MHz, CDCl_3_): δ 14.39, 64.90, 111.96, 119.88, 123.50, 125.48, 129.74, 130.82, 133.44, 144.19, 150.06, 156.77, 197.28; IR (KBr cm^−1^): 1662 (C=O), 1603, 1515, 1472 (Ar, naphthalene), 1229 (=C—O—C); HRMS (*m*/*z*): [*M* + H]^+^ Calculated for C_28_H_22_N_2_O_8_, 515.1410; found, 515.1449; m.p. = 556.4–568.5 K.

## Refinement   

Crystal data, data collection and structure refinement details are summarized in Table 2 ▶. All H atoms were located in a difference Fourier map and were subsequently refined as riding atoms, with C—H = 0.95 (aromatic), 0.98 (meth­yl) and 0.99 Å (methyl­ene), and with *U*_iso_(H) = 1.2*U*_eq_(C). The positions of methyl H atoms were rotationally optimized.

## Supplementary Material

Crystal structure: contains datablock(s) I. DOI: 10.1107/S1600536814018674/is5370sup1.cif

Structure factors: contains datablock(s) I. DOI: 10.1107/S1600536814018674/is5370Isup2.hkl

Supporting information file. DOI: 10.1107/S1600536814018674/is5370Isup3.pdf

Supporting information file. DOI: 10.1107/S1600536814018674/is5370Isup4.pdf

Supporting information file. DOI: 10.1107/S1600536814018674/is5370Isup5.pdf

Supporting information file. DOI: 10.1107/S1600536814018674/is5370Isup6.cml

CCDC reference: 1019755

Additional supporting information:  crystallographic information; 3D view; checkCIF report

## Figures and Tables

**Table 1:** Hydrogen-bond geometry (Å, °) *Cg*1 and *Cg*2 are the centroids of the six-membered C1–C6 and C1^v^–C4^v^/C5/C6 rings, respectively. [Symmetry code: (v) −*x* + 1, *y*, −*z* + 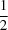.]

*D*—H⋯*A*	*D*—H	H⋯*A*	*D*⋯*A*	*D*—H⋯*A*
C13—H13⋯O1^i^	0.95	2.50	3.2129 (17)	132
C10—H10⋯O3^ii^	0.95	2.59	3.360 (2)	138
C14—H14*B*⋯*Cg*1^iii^	0.99	2.81	3.6284 (15)	140
C14—H14*B*⋯*Cg*2^iv^	0.99	2.81	3.6284 (15)	140

Symmetry codes: (i) 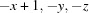; (ii) 
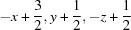
; (iii) 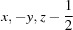; (iv) 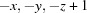.

**Table 2:** Experimental details

Crystal data	
Chemical formula	C_28_H_22_N_2_O_8_
*M*_r_	514.48
Crystal system, space group	Monoclinic, *C*2/*c*
Temperature (K)	193
*a*, *b*, *c* (Å)	23.5359 (16), 10.2522 (5), 10.3605 (11)
β (°)	97.257 (14)
*V* (Å^3^)	2479.9 (3)
*Z*	4
Radiation type	Cu *K*α
μ (mm^−1^)	0.86
Crystal size (mm)	0.50 × 0.40 × 0.20

Data collection	
Diffractometer	Rigaku R-AXIS RAPID
Absorption correction	Numerical (*NUMABS*; Higashi, 1999 ▶)
*T*_min_, *T*_max_	0.674, 0.847
No. of measured, independent and observed [*I* > 2σ(*I*)] reflections	21420, 2272, 2118
*R*_int_	0.044
(sin θ/λ)_max_ (Å^−1^)	0.602

Refinement	
*R*[*F* ^2^ > 2σ(*F* ^2^)], *wR*(*F* ^2^), *S*	0.037, 0.101, 1.02
No. of reflections	2272
No. of parameters	175
H-atom treatment	H-atom parameters constrained
Δρ_max_, Δρ_min_ (e Å^−3^)	0.21, −0.26

Computer programs: *PROCESS-AUTO* (Rigaku, 1998 ▶), *CrystalStructure* (Rigaku, 2010 ▶), *SIR2004* (Burla *et al.*, 2007 ▶), *SHELXL97* (Sheldrick, 2008 ▶) and *ORTEPIII* (Burnett & Johnson, 1996 ▶).

## supplementary crystallographic information

### Crystal data

**Table:**

C_28_H_22_N_2_O_8_	*F*(000) = 1072
*M**_r_* = 514.48	*D*_x_ = 1.378 Mg m^−^^3^
Monoclinic, *C*2/*c*	Cu *K*α radiation, λ = 1.54187 Å
Hall symbol: -C 2yc	Cell parameters from 1640 reflections
*a* = 23.5359 (16) Å	θ = 3.8–67.5°
*b* = 10.2522 (5) Å	µ = 0.86 mm^−^^1^
*c* = 10.3605 (11) Å	*T* = 193 K
β = 97.257 (14)°	Block, yellow
*V* = 2479.9 (3) Å^3^	0.50 × 0.40 × 0.20 mm
*Z* = 4	

### Data collection

**Table:**

Rigaku R-AXIS RAPID diffractometer	2272 independent reflections
Radiation source: rotating anode	2118 reflections with *I* > 2σ(*I*)
Graphite monochromator	*R*_int_ = 0.044
Detector resolution: 10.000 pixels mm^-1^	θ_max_ = 68.2°, θ_min_ = 3.8°
ω scans	*h* = −28→27
Absorption correction: numerical (*NUMABS*; Higashi, 1999)	*k* = −12→12
*T*_min_ = 0.674, *T*_max_ = 0.847	*l* = −12→12
21420 measured reflections	

### Refinement

**Table:**

Refinement on *F*^2^	Secondary atom site location: difference Fourier map
Least-squares matrix: full	Hydrogen site location: inferred from neighbouring sites
*R*[*F*^2^ > 2σ(*F*^2^)] = 0.037	H-atom parameters constrained
*wR*(*F*^2^) = 0.101	*w* = 1/[σ^2^(*F*_o_^2^) + (0.0552*P*)^2^ + 1.320*P*] where *P* = (*F*_o_^2^ + 2*F*_c_^2^)/3
*S* = 1.02	(Δ/σ)_max_ = 0.001
2272 reflections	Δρ_max_ = 0.21 e Å^−^^3^
175 parameters	Δρ_min_ = −0.26 e Å^−^^3^
0 restraints	Extinction correction: *SHELXL97* (Sheldrick, 2008), Fc^*^=kFc[1+0.001xFc^2^λ^3^/sin(2θ)]^-1/4^
Primary atom site location: structure-invariant direct methods	Extinction coefficient: 0.0031 (2)

### Special details

**Table:**

Geometry. All e.s.d.'s (except the e.s.d. in the dihedral angle between two l.s. planes) are estimated using the full covariance matrix. The cell e.s.d.'s are taken into account individually in the estimation of e.s.d.'s in distances, angles and torsion angles; correlations between e.s.d.'s in cell parameters are only used when they are defined by crystal symmetry. An approximate (isotropic) treatment of cell e.s.d.'s is used for estimating e.s.d.'s involving l.s. planes.
Refinement. Refinement of *F*^2^ against ALL reflections. The weighted *R*-factor *wR* and goodness of fit *S* are based on *F*^2^, conventional *R*-factors *R* are based on *F*, with *F* set to zero for negative *F*^2^. The threshold expression of *F*^2^ > σ(*F*^2^) is used only for calculating *R*-factors(gt) *etc*. and is not relevant to the choice of reflections for refinement. *R*-factors based on *F*^2^ are statistically about twice as large as those based on *F*, and *R*- factors based on ALL data will be even larger.

### Fractional atomic coordinates and isotropic or equivalent isotropic displacement parameters (Å^2^)

**Table:**

	*x*	*y*	*z*	*U*_iso_*/*U*_eq_	
O1	0.48373 (3)	0.18553 (8)	0.10345 (8)	0.0365 (2)	
O2	0.60134 (4)	0.37631 (9)	0.01960 (9)	0.0428 (3)	
O3	0.72391 (7)	−0.19473 (16)	0.10952 (14)	0.0991 (6)	
O4	0.76491 (5)	−0.08350 (14)	0.27145 (16)	0.0812 (4)	
N1	0.72522 (6)	−0.10377 (14)	0.18632 (14)	0.0584 (4)	
C1	0.53601 (5)	0.37563 (12)	0.16932 (11)	0.0305 (3)	
C2	0.57104 (5)	0.44716 (13)	0.09761 (11)	0.0347 (3)	
C3	0.57153 (5)	0.58479 (13)	0.10108 (12)	0.0397 (3)	
H3	0.5962	0.6327	0.0528	0.048*	
C4	0.53620 (5)	0.64790 (13)	0.17435 (12)	0.0396 (3)	
H4	0.5359	0.7406	0.1746	0.048*	
C5	0.5000	0.58029 (16)	0.2500	0.0346 (4)	
C6	0.5000	0.44053 (16)	0.2500	0.0303 (4)	
C7	0.53051 (5)	0.23260 (12)	0.14003 (11)	0.0306 (3)	
C8	0.58275 (5)	0.14797 (11)	0.15282 (11)	0.0325 (3)	
C9	0.63069 (5)	0.17560 (12)	0.24136 (12)	0.0368 (3)	
H9	0.6313	0.2522	0.2932	0.044*	
C10	0.67737 (5)	0.09281 (13)	0.25474 (13)	0.0415 (3)	
H10	0.7099	0.1102	0.3164	0.050*	
C11	0.67537 (6)	−0.01629 (13)	0.17554 (14)	0.0422 (3)	
C12	0.62879 (6)	−0.04532 (13)	0.08520 (14)	0.0455 (3)	
H12	0.6288	−0.1204	0.0314	0.055*	
C13	0.58214 (6)	0.03741 (13)	0.07495 (13)	0.0410 (3)	
H13	0.5494	0.0187	0.0143	0.049*	
C14	0.64361 (6)	0.43699 (15)	−0.04805 (14)	0.0458 (3)	
H14A	0.6698	0.4913	0.0121	0.055*	
H14B	0.6252	0.4930	−0.1193	0.055*	
C15	0.67573 (7)	0.32734 (18)	−0.10160 (18)	0.0615 (4)	
H15A	0.6952	0.2756	−0.0297	0.074*	
H15B	0.7041	0.3631	−0.1535	0.074*	
H15C	0.6488	0.2717	−0.1567	0.074*	

### Atomic displacement parameters (Å^2^)

**Table:**

	*U*^11^	*U*^22^	*U*^33^	*U*^12^	*U*^13^	*U*^23^
O1	0.0325 (5)	0.0357 (5)	0.0403 (5)	−0.0042 (3)	0.0003 (3)	−0.0047 (4)
O2	0.0451 (5)	0.0403 (5)	0.0458 (5)	−0.0028 (4)	0.0167 (4)	0.0027 (4)
O3	0.1189 (12)	0.1059 (12)	0.0720 (9)	0.0757 (10)	0.0100 (8)	−0.0127 (8)
O4	0.0517 (7)	0.0739 (9)	0.1132 (11)	0.0221 (6)	−0.0077 (7)	0.0084 (8)
N1	0.0580 (8)	0.0581 (8)	0.0623 (8)	0.0231 (6)	0.0198 (7)	0.0154 (7)
C1	0.0301 (6)	0.0293 (6)	0.0306 (6)	−0.0002 (4)	−0.0027 (5)	0.0010 (4)
C2	0.0338 (6)	0.0353 (7)	0.0338 (6)	−0.0014 (5)	−0.0004 (5)	0.0012 (5)
C3	0.0414 (7)	0.0351 (7)	0.0418 (7)	−0.0071 (5)	0.0024 (5)	0.0071 (5)
C4	0.0463 (7)	0.0276 (6)	0.0427 (7)	−0.0031 (5)	−0.0033 (6)	0.0033 (5)
C5	0.0370 (9)	0.0294 (9)	0.0349 (8)	0.000	−0.0050 (7)	0.000
C6	0.0304 (8)	0.0282 (8)	0.0300 (8)	0.000	−0.0047 (6)	0.000
C7	0.0327 (6)	0.0323 (6)	0.0266 (5)	−0.0025 (5)	0.0024 (4)	−0.0004 (5)
C8	0.0334 (6)	0.0291 (6)	0.0354 (6)	−0.0036 (5)	0.0058 (5)	−0.0004 (5)
C9	0.0364 (6)	0.0340 (7)	0.0393 (6)	−0.0015 (5)	0.0024 (5)	−0.0027 (5)
C10	0.0346 (7)	0.0444 (8)	0.0447 (7)	0.0002 (5)	0.0017 (5)	0.0042 (6)
C11	0.0394 (7)	0.0388 (7)	0.0507 (7)	0.0082 (5)	0.0140 (6)	0.0090 (6)
C12	0.0502 (8)	0.0351 (7)	0.0528 (8)	0.0026 (6)	0.0129 (6)	−0.0074 (6)
C13	0.0385 (7)	0.0377 (7)	0.0464 (7)	−0.0029 (5)	0.0036 (5)	−0.0085 (6)
C14	0.0384 (7)	0.0536 (8)	0.0463 (7)	−0.0059 (6)	0.0094 (6)	0.0084 (6)
C15	0.0469 (8)	0.0731 (11)	0.0686 (10)	0.0035 (7)	0.0238 (7)	0.0044 (8)

### Geometric parameters (Å, º)

**Table:**

O1—C7	1.2181 (14)	C7—C8	1.4970 (16)
O2—C2	1.3546 (15)	C8—C13	1.3903 (17)
O2—C14	1.4298 (15)	C8—C9	1.3904 (17)
O3—N1	1.224 (2)	C9—C10	1.3813 (18)
O4—N1	1.2188 (19)	C9—H9	0.9500
N1—C11	1.4698 (17)	C10—C11	1.385 (2)
C1—C2	1.3872 (17)	C10—H10	0.9500
C1—C6	1.4278 (14)	C11—C12	1.381 (2)
C1—C7	1.4997 (17)	C12—C13	1.3809 (19)
C2—C3	1.4116 (19)	C12—H12	0.9500
C3—C4	1.3584 (19)	C13—H13	0.9500
C3—H3	0.9500	C14—C15	1.500 (2)
C4—C5	1.4105 (15)	C14—H14A	0.9900
C4—H4	0.9500	C14—H14B	0.9900
C5—C4^i^	1.4105 (15)	C15—H15A	0.9800
C5—C6	1.433 (2)	C15—H15B	0.9800
C6—C1^i^	1.4278 (14)	C15—H15C	0.9800
			
C2—O2—C14	120.70 (11)	C10—C9—C8	120.65 (12)
O4—N1—O3	123.69 (14)	C10—C9—H9	119.7
O4—N1—C11	118.84 (14)	C8—C9—H9	119.7
O3—N1—C11	117.46 (15)	C9—C10—C11	118.03 (12)
C2—C1—C6	120.28 (12)	C9—C10—H10	121.0
C2—C1—C7	116.77 (10)	C11—C10—H10	121.0
C6—C1—C7	122.12 (11)	C12—C11—C10	122.71 (12)
O2—C2—C1	115.43 (11)	C12—C11—N1	118.54 (13)
O2—C2—C3	123.23 (11)	C10—C11—N1	118.75 (13)
C1—C2—C3	121.22 (12)	C11—C12—C13	118.37 (12)
C4—C3—C2	119.13 (12)	C11—C12—H12	120.8
C4—C3—H3	120.4	C13—C12—H12	120.8
C2—C3—H3	120.4	C12—C13—C8	120.41 (12)
C3—C4—C5	122.13 (12)	C12—C13—H13	119.8
C3—C4—H4	118.9	C8—C13—H13	119.8
C5—C4—H4	118.9	O2—C14—C15	105.65 (12)
C4^i^—C5—C4	121.13 (16)	O2—C14—H14A	110.6
C4^i^—C5—C6	119.43 (8)	C15—C14—H14A	110.6
C4—C5—C6	119.43 (8)	O2—C14—H14B	110.6
C1—C6—C1^i^	124.45 (15)	C15—C14—H14B	110.6
C1—C6—C5	117.77 (7)	H14A—C14—H14B	108.7
C1^i^—C6—C5	117.77 (7)	C14—C15—H15A	109.5
O1—C7—C1	120.12 (10)	C14—C15—H15B	109.5
O1—C7—C8	119.87 (11)	H15A—C15—H15B	109.5
C1—C7—C8	120.00 (9)	C14—C15—H15C	109.5
C13—C8—C9	119.81 (12)	H15A—C15—H15C	109.5
C13—C8—C7	118.22 (11)	H15B—C15—H15C	109.5
C9—C8—C7	121.96 (11)		
			
C14—O2—C2—C1	−173.03 (10)	C2—C1—C7—C8	58.11 (14)
C14—O2—C2—C3	10.93 (18)	C6—C1—C7—C8	−132.34 (10)
C6—C1—C2—O2	−176.93 (8)	O1—C7—C8—C13	26.83 (17)
C7—C1—C2—O2	−7.18 (14)	C1—C7—C8—C13	−151.79 (11)
C6—C1—C2—C3	−0.81 (16)	O1—C7—C8—C9	−151.86 (12)
C7—C1—C2—C3	168.94 (11)	C1—C7—C8—C9	29.52 (17)
O2—C2—C3—C4	174.63 (11)	C13—C8—C9—C10	−1.34 (19)
C1—C2—C3—C4	−1.18 (18)	C7—C8—C9—C10	177.33 (11)
C2—C3—C4—C5	1.68 (18)	C8—C9—C10—C11	1.42 (19)
C3—C4—C5—C4^i^	179.81 (13)	C9—C10—C11—C12	−0.4 (2)
C3—C4—C5—C6	−0.19 (13)	C9—C10—C11—N1	178.57 (12)
C2—C1—C6—C1^i^	−177.76 (11)	O4—N1—C11—C12	−175.30 (14)
C7—C1—C6—C1^i^	13.05 (7)	O3—N1—C11—C12	3.6 (2)
C2—C1—C6—C5	2.24 (11)	O4—N1—C11—C10	5.7 (2)
C7—C1—C6—C5	−166.95 (7)	O3—N1—C11—C10	−175.35 (14)
C4^i^—C5—C6—C1	178.24 (8)	C10—C11—C12—C13	−0.8 (2)
C4—C5—C6—C1	−1.76 (8)	N1—C11—C12—C13	−179.72 (12)
C4^i^—C5—C6—C1^i^	−1.76 (8)	C11—C12—C13—C8	0.9 (2)
C4—C5—C6—C1^i^	178.24 (8)	C9—C8—C13—C12	0.2 (2)
C2—C1—C7—O1	−120.50 (12)	C7—C8—C13—C12	−178.56 (12)
C6—C1—C7—O1	49.05 (15)	C2—O2—C14—C15	169.06 (11)

Symmetry code: (i) −*x*+1, *y*, −*z*+1/2.

### Hydrogen-bond geometry (Å, º)

*Cg*1 and *Cg*2 are the centroids of the six-membered C1–C6 and C1^v^–C4^v^/C5/C6 rings, respectively. [Symmetry code: (v) -*x*+1, *y*, -*z*+1/2.]

**Table:**

*D*—H···*A*	*D*—H	H···*A*	*D*···*A*	*D*—H···*A*
C13—H13···O1^ii^	0.95	2.50	3.2129 (17)	132
C10—H10···O3^iii^	0.95	2.59	3.360 (2)	138
C14—H14*B*···*Cg*1^iv^	0.99	2.81	3.6284 (15)	140
C14—H14*B*···*Cg*2^v^	0.99	2.81	3.6284 (15)	140

Symmetry codes: (ii) −*x*+1, −*y*, −*z*; (iii) −*x*+3/2, *y*+1/2, −*z*+1/2; (iv) *x*, −*y*, *z*−1/2; (v) −*x*, −*y*, −*z*+1.

## References

[bb1] Allen, F. H. (2002). *Acta Cryst.* B**58**, 380–388. 10.1107/s0108768102003890 12037359

[bb2] Bulman Page, P. C., Bartlett, C. J., Chan, Y., Day, D., Parker, P., Buckley, B. R., Rassias, G. A., Slawin, A. M. Z., Allin, S. M. & Lacour, J. (2012). *J. Org. Chem.* **77**, 6128–6138. 10.1021/jo300915u 22708806

[bb3] Burla, M. C., Caliandro, R., Camalli, M., Carrozzini, B., Cascarano, G. L., De Caro, L., Giacovazzo, C., Polidori, G., Siliqi, D. & Spagna, R. (2007). *J. Appl. Cryst.* **40**, 609–613.

[bb4] Burnett, M. N. & Johnson, C. K. (1996). *ORTEPIII* Report ORNL-6895. Oak Ridge National Laboratory. Tennessee, USA.

[bb5] Cohen, S., Thirumalaikumar, M., Pogodin, S. & Agranat, I. (2004). *Struct. Chem.* **15**, 339–346.

[bb6] Hatano, S., Horino, T., Tokita, A., Oshima, T. & Abe, J. (2013). *J. Am. Chem. Soc.* **135**, 3164–3172. 10.1021/ja311344u 23402262

[bb7] Higashi, T. (1999). *NUMABS* Rigaku Corporation, Tokyo, Japan.

[bb8] Isogai, A., Tsumuki, T., Murohashi, S., Okamoto, A. & Yonezawa, N. (2013). *Acta Cryst.* E**69**, o71. 10.1107/S1600536812049963 PMC3588252 23476452

[bb9] Jayalakshmi, V., Wood, T., Basu, R., Du, J., Blackburn, T., Rosenblatt, C., Crudden, C. M. & Lemieux, R. P. (2012). *J. Mater. Chem.* **22**, 15255–15261.

[bb10] Jing, L.-H., Qin, D.-B., He, L., Gu, S.-J., Zhang, H.-X. & Lei, G. (2005). *Acta Cryst.* E**61**, o3595–o3596.

[bb11] Kano, T., Tokuda, O. & Maruoka, K. (2006). *Tetrahedron Lett.* **47**, 7423–7426.

[bb12] Nakaema, K., Watanabe, S., Okamoto, A., Noguchi, K. & Yonezawa, N. (2008). *Acta Cryst.* E**64**, o807. 10.1107/S1600536808007009 PMC2961268 21202298

[bb13] Okamoto, A., Mitsui, R., Oike, H. & Yonezawa, N. (2011). *Chem. Lett.* **40**, 1283–1284.

[bb14] Okamoto, A. & Yonezawa, N. (2009). *Chem. Lett.* **38**, 914–915.

[bb15] Park, J. K., Lee, K. H., Park, J. S., Seo, J. H., Kim, Y. K. & Yoon, S. S. (2010). *Mol. Cryst. Liq. Cryst.* **531**, 55–64.

[bb16] Rigaku (1998). *PROCESS-AUTO* Rigaku Corporation, Tokyo, Japan.

[bb17] Rigaku (2010). *CrystalStructure* Rigaku Corporation, Tokyo, Japan.

[bb18] Sheldrick, G. M. (2008). *Acta Cryst.* A**64**, 112–122. 10.1107/S0108767307043930 18156677

[bb19] Vaghi, L., Benincori, T., Cirilli, R., Alberico, E., Mussini, P., Romana, P., Marco, P. T., Rizzo, S. & Sannicolo, F. (2013). *Eur. J. Org. Chem.* pp. 8174–8184.

[bb20] Wang, Y., McGonigal, P. R., Herle, B., Basora, M. & Echavarren, A. (2014). *J. Am. Chem. Soc.* **136**, 801–809. 10.1021/ja411626v PMC3898715 24358986

[bb21] Watanabe, S., Nakaema, K., Nishijima, T., Okamoto, A. & Yonezawa, N. (2010). *Acta Cryst.* E**66**, o615. 10.1107/S1600536810005398 PMC2983576 21580373

