# Supplementary material for: Crystal structure of 2,7-dieth­oxy-1,8-bis­(4-nitro­benzo­yl)naphthalene
Source: Acta Crystallogr Sect E Struct Rep Online. 2014 Aug 23;70(Pt 9):138–41. doi: 10.1107/S1600536814018674 (PMC4186128; doi:10.1107/S1600536814018674)
Supplement: Supplementary file 3 [file e-70-00138-Isup3.pdf]

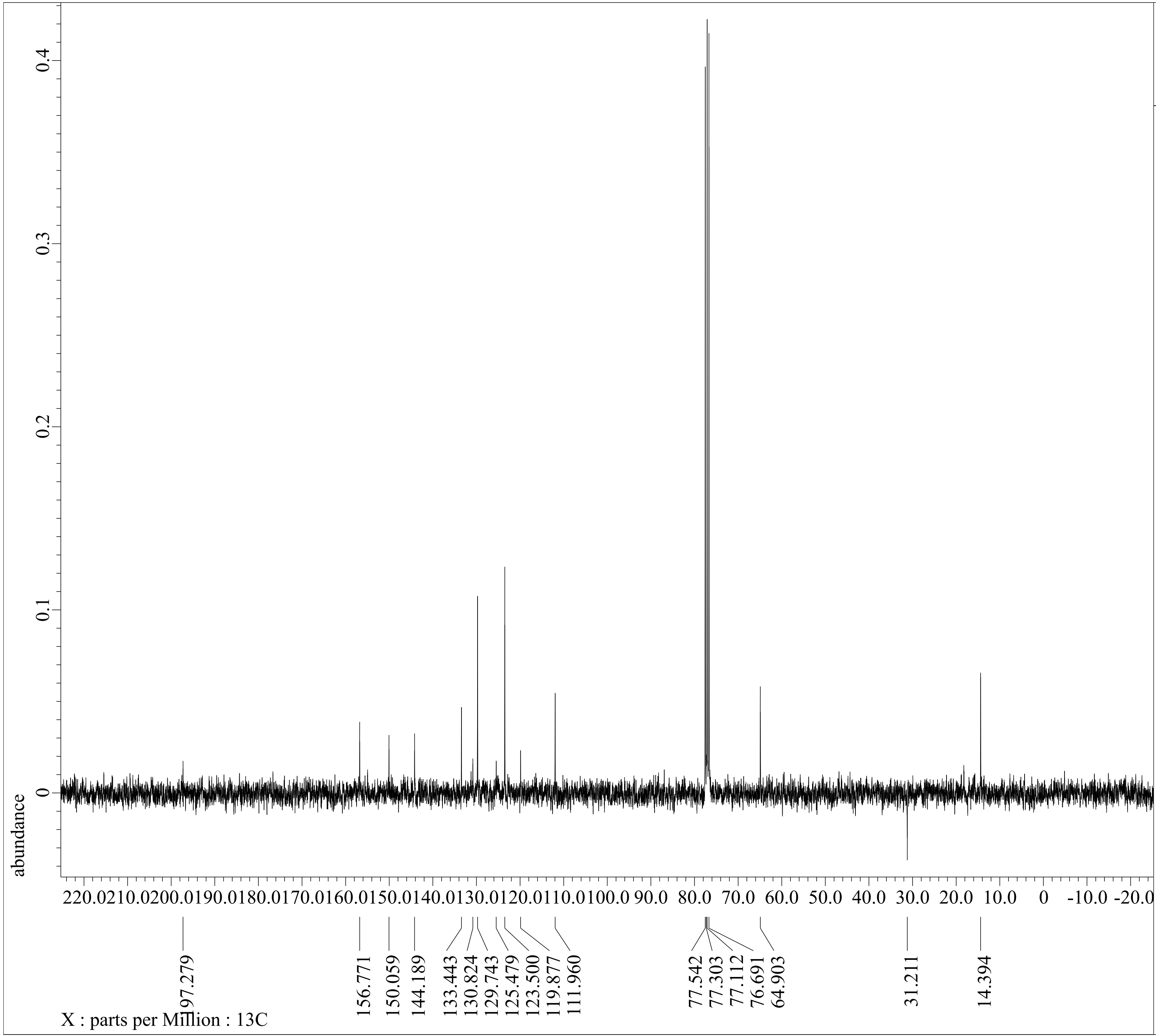

Filename = /Volumes/Untitled/NMR/carb
Author = delta
Experiment = single_pulse_dec
Sample_Id = S#647291
Solvent = CHLOROFORM-D
Creation_Time = 14-JUL-2014 17:37:34
Revision_Time = 14-JUL-2014 19:00:59
Current_Time = 17-JUL-2014 18:59:36

Comment = single pulse decoupled gat
Data_Format = 1D COMPLEX
Dim_Size = 26214
Dim_Title = 13C
Dim_Units = [ppm]
Dimensions = X
Site = ECX 300
Spectrometer = JNM-ECX300

Field_Strength = 7.0586013[T] (300[MHz])
X_Acq_Duration = 1.38412032[s]
X_Domain = 13C
X_Freq = 75.56823426[MHz]
X_Offset = 100[ppm]
X_Points = 32768
X_Prescans = 4
X_Resolution = 0.72248054[Hz]
X_Sweep = 23.67424242[kHz]
Irr_Domain = 1H
Irr_Freq = 300.52965592[MHz]
Irr_Offset = 5[ppm]
Clipped = FALSE
Scans = 159
Total_Scans = 159

Relaxation_Delay = 2[s]
Recvr_Gain = 60
Temp_Get = 22[dC]
X_90_Width = 9.5[us]
X_Acq_Time = 1.38412032[s]
X_Angle = 30[deg]
X_Atn = 5.3[dB]
X_Pulse = 3.16666667[us]
Irr_Atn_Dec = 21.35[dB]
Irr_Atn_Noe = 21.35[dB]
Irr_Noise = WALTZ
Decoupling = TRUE
Initial_Wait = 1[s]
Noe = TRUE
Noe_Time = 2[s]
Repetition_Time = 3.38412032[s]
