# Supplementary material for: Crystal structure of 2,7-dieth­oxy-1,8-bis­(4-nitro­benzo­yl)naphthalene
Source: Acta Crystallogr Sect E Struct Rep Online. 2014 Aug 23;70(Pt 9):138–41. doi: 10.1107/S1600536814018674 (PMC4186128; doi:10.1107/S1600536814018674)
Supplement: Supplementary file 4 [file e-70-00138-Isup4.pdf]

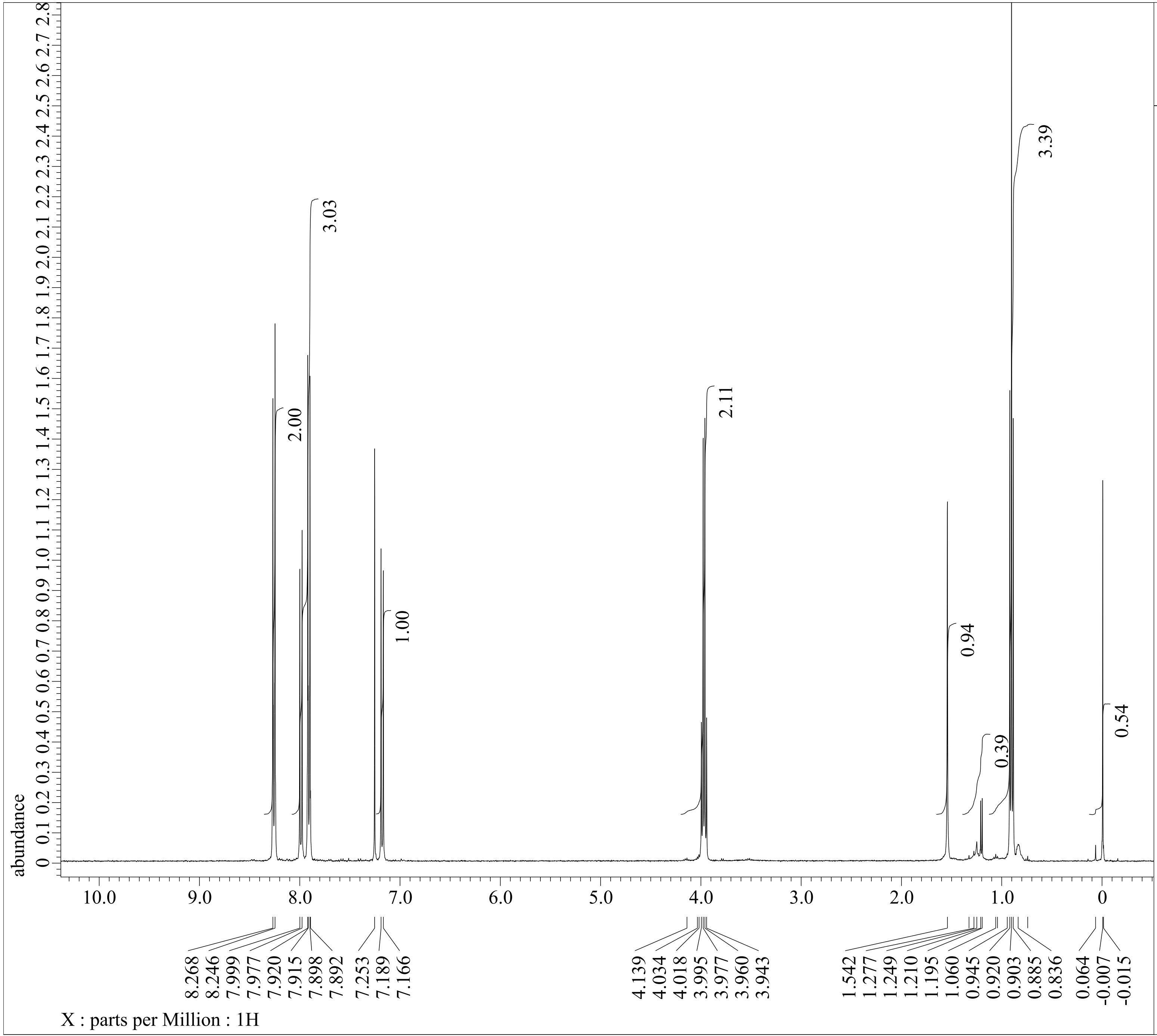

Filename	=	/Volumes/Untitled/NMR/AUTH
Author	=	delta
Experiment	=	single_pulse.ex2
Sample_Id	=	S#643639
Solvent	=	CHLOROFORM-D
Creation_Time	=	23-AUG-2012 15:26:53
Revision_Time	=	17-JUL-2014 19:11:31
Current_Time	=	17-JUL-2014 19:12:38
Comment	=	single_pulse
Data_Format	=	1D COMPLEX
Dim_Size	=	13107
Dim_Title	=	1H
Dim_Units	=	[ppm]
Dimensions	=	X
Site	=	ECX 400
Spectrometer	=	JNM-ECX400
Field_Strength	=	9.389766[T] (400[MHz])
X_Acq_Duration	=	2.18365952[s]
X_Domain	=	1H
X_Freq	=	399.78219838[MHz]
X_Offset	=	5[ppm]
X_Points	=	16384
X_Prescans	=	1
X_Resolution	=	0.45794685[Hz]
X_Sweep	=	7.5030012[kHz]
Irr_Domain	=	1H
Irr_Freq	=	399.78219838[MHz]
Irr_Offset	=	5[ppm]
Tri_Domain	=	1H
Tri_Freq	=	399.78219838[MHz]
Tri_Offset	=	5[ppm]
Clipped	=	FALSE
Scans	=	4
Total_Scans	=	4
Relaxation_Delay	=	5[s]
Recvr_Gain	=	42
Temp_Get	=	25.8[dC]
X_90_Width	=	12.25[us]
X_Acq_Time	=	2.18365952[s]
X_Angle	=	45[deg]
X_Atn	=	4.5[dB]
X_Pulse	=	6.125[us]
Irr_Mode	=	Off
Tri_Mode	=	Off
Dante_Presat	=	FALSE
Initial_Wait	=	1[s]
Repetition_Time	=	7.18365952[s]
