# Supplementary material for: Crystal structure of 2,7-dieth­oxy-1,8-bis­(4-nitro­benzo­yl)naphthalene
Source: Acta Crystallogr Sect E Struct Rep Online. 2014 Aug 23;70(Pt 9):138–41. doi: 10.1107/S1600536814018674 (PMC4186128; doi:10.1107/S1600536814018674)
Supplement: Supplementary file 5 [file e-70-00138-Isup5.pdf]

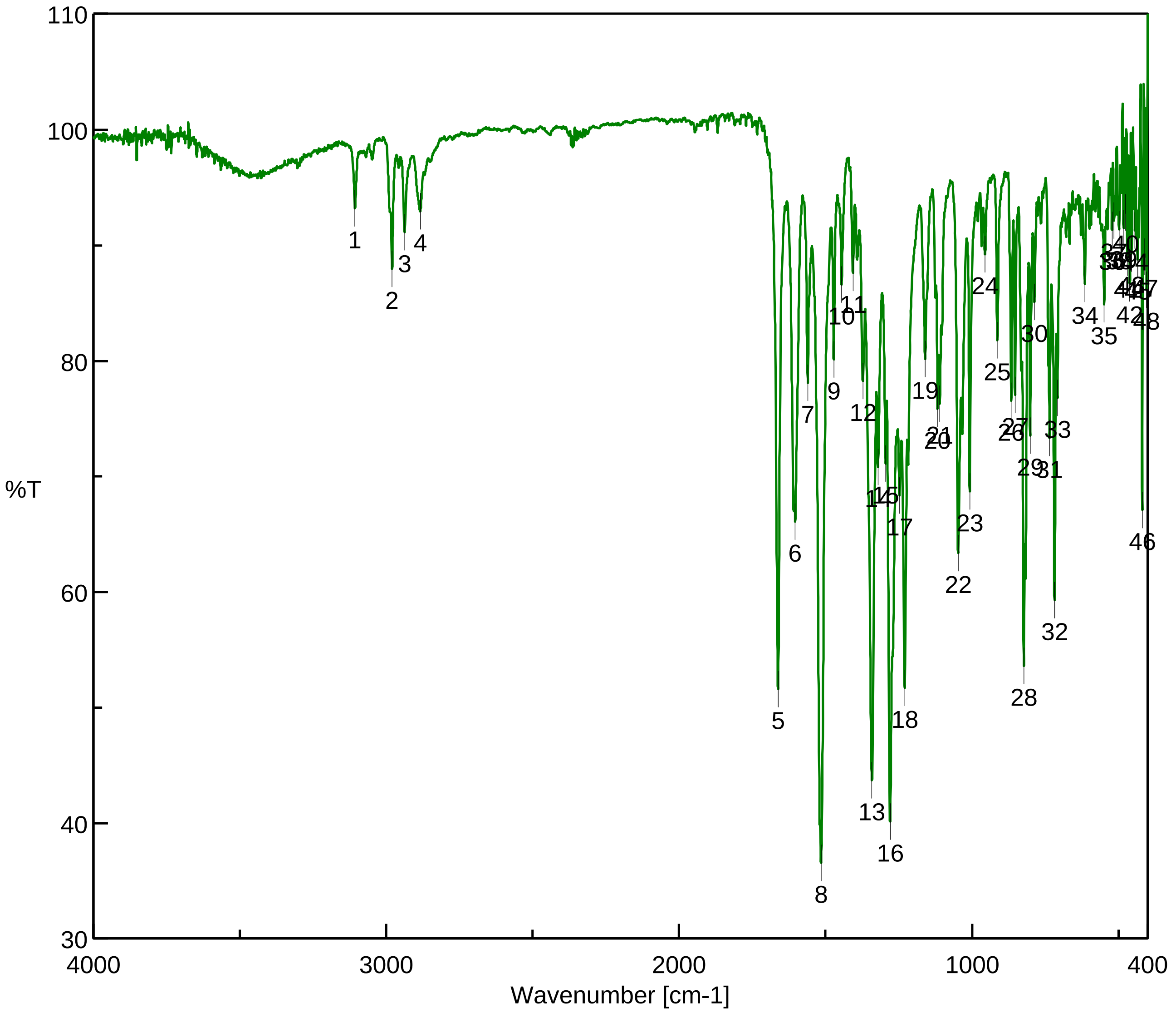

ピーク検出結果

No.	位置	強度	No.	位置	強度
1	3106.76	93.1654	2	2979.96	87.9321
3	2937.54	91.118	4	2884.02	92.914
5	1662.34	51.5947	6	1603.04	66.0706
7	1560.61	78.0514	8	1515.29	36.551
9	1471.9	80.0677	10	1444.9	86.5842
11	1406.33	87.563	12	1372.59	78.2359
13	1341.25	43.6758	14	1320.52	70.7596
15	1294.97	71.0995	16	1279.54	40.0954
17	1247.72	68.3079	18	1229.88	51.6885
19	1159.97	80.1189	20	1117.06	75.7953
21	1109.83	76.28	22	1047.16	63.3602
23	1007.62	68.6516	24	955.073	89.1712
25	913.611	81.7508	26	865.4	76.5153
27	852.865	77.0179	28	822.973	53.5935
29	801.278	73.5086	30	786.332	85.0781
31	734.746	73.2953	32	717.872	59.2876
33	708.229	76.7616	34	614.217	86.6058
35	548.649	84.877	36	521.65	91.2822
37	514.901	92.0896	38	497.544	91.346
39	482.117	91.4315	40	474.885	92.7935
41	469.582	88.8979	42	460.904	86.6849
43	454.636	89.226	44	443.065	91.2218
45	434.387	88.7234	46	418.477	67.0617
47	410.281	88.962	48	403.05	86.1189

[コメント情報]

試料名	
コメント	
測定者	
所属	
会社	東京農工大学

[測定情報]

機種名	FT/IR-4100typeA
シリアル番号	B041461016
光源	標準光源
検出器	TGS
積算回数	16
分解	2 cm−1
ゼロフィリング	On
アポダイゼーション	Cosine
ゲイン	Auto (8)
アパーチャー	Auto (5 mm)
スキャンスピード	Auto (2 mm/sec)
フィルタ	Auto (30000 Hz)
